# Association Between Food Patterns and Gray Matter Volume

**DOI:** 10.3389/fnhum.2019.00384

**Published:** 2019-10-29

**Authors:** Keisuke Kokubun, Yoshinori Yamakawa

**Affiliations:** ^1^Office of Society-Academia Collaboration for Innovation, Kyoto University, Kyoto, Japan; ^2^ImPACT Program of Council for Science, Technology and Innovation (Cabinet Office, Government of Japan), Chiyoda, Japan; ^3^Institute of Innovative Research, Tokyo Institute of Technology, Meguro, Japan; ^4^NTT Data Institute of Management Consulting, Inc., Minato, Japan

**Keywords:** normalized gray matter volume, brief self-administered diet history questionnaire, food patterns, magnetic resonance imaging, alcohol and animal foods dietary pattern, vegetable-animal balanced dietary pattern

## Abstract

Diet and nutrition play a key role in the promotion and maintenance of good health, as they are important modifiable risk factors for chronic diseases. A growing number of studies indicate that optimal food intake and optimal physical activity are essential for the gray matter volume (GMV). However, the precise definition of “optimal” is extremely difficult and a topic of several studies. In the current research, we used the magnetic resonance imaging (MRI)-based normalized GMV (nGMV), for monitoring brain conditions based on GMV. By analyzing the relationship between the nGMV of 171 healthy Japanese participants and the results of a brief self-administered diet history questionnaire (BDHQ), we found that while nGMV was high in the participants with high intake of milk and yogurt, it was low in the participants of “alcohol and animal foods dietary pattern” (high intake of alcohol and animal foods). On the other hand, another food pattern “vegetable-animal balanced dietary pattern” (balanced intake of vegetables and animal foods) has no significant association with nGMV, indicating that although a diet consisting of a good balance of vegetables and animal foods may not lead to brain atrophy, it might not positively contribute to a higher nGMV. nGMV, as an objective measure of the association between food intake and the brain, might provide useful information for “optimal” food intake for GMV.

## Introduction

One’s food choices can make a big difference to one’s health and wellbeing. Moreover, growing number of studies indicate that optimal foods and nutrition are essential for brain conditions as determined by gray matter volume (GMV) (Titova et al., 2013b; Pelletier et al., 2015; Gu et al., 2016; Croll et al., 2018). However, a precise definition of “optimal” is extremely difficult and is being extensively studied (Dauncey, 2012).

Prior studies have examined the association between GMV and foods such as fish (Raji et al., 2014), fish oil (Witte et al., 2013), and meat products (Titova et al., 2013a); however, the results have been inconsistent. The investigation of a single food factor in relation to the brain volume may be difficult, given that foods are consumed in combination and their complex effects are likely to be interactive or synergistic (Hu, 2002). To overcome these issues, the analysis of dietary patterns has gained much interest because a dietary pattern is a comprehensive variable that integrates consumption of several foods or food groups and is expected to have a greater impact on human health than any single nutrient (Hu, 2002).

For instance, some studies, such as Jacka et al. (2015) have used a data-driven, latent factor approach to determine diet composition groups, and then looked at longitudinal changes in the hippocampus in late middle-aged adults (i.e., 60–64 years). Espeland et al. (2016) in middle-aged to aged adults showed less total white matter hyperintensity burden after a broad “healthy lifestyle” intervention that partly included information on diet. By contrast, most of this literature has focused on a specific dietary pattern, the Mediterranean (Medi) diet, and brain volumes in aged, non-demented adults (i.e., 65+ years of age). Exceptionally, a study in Japan showed that the type of breakfast (rice or bread) affected brain gray and white matter volumes and cognitive function in healthy children (Taki et al., 2010). However, we are not aware of any study on the relationship between general dietary patterns (not only Medi diet but also more diverse foods) and brain volume among healthy adults (including middle aged, 30s to 60s years old).

In our previous study, we developed the MRI-based normalized gray matter volume (nGMV), which is approved as the international standard as per the recommendation ITU-TH.861.1 by the International Telecommunication Union Telecommunication (ITU-T) Standardization Sector, for monitoring brain conditions based on GMV (ITU-T, 2018). The nGMV employs a standardized score (e.g., mean of population is 100 and 1 standard deviation is 15, so that 95% of population falls within the range of 70–130) calculated by averaging individual regions’ scores. Therefore, it could be said that the nGMV has biological characteristics (Porter, 2004). On the other hand, recent algorithms derived from deep learning are for estimating objective variables and lack such biological features. To sum up, they have a mutually complementary relationship. Moreover, we also showed that nGMV is inversely correlated with age and BMI (Nemoto et al., 2017) and could be a means to objectively evaluate stress and fatigue (Kokubun et al., 2018). These results are in line with the growing number of research which has indicated the existence of positive correlations of regional GMV with material factors of healthy life including emotional intelligence (Killgore et al., 2012), affective and cognitive empathy (Eres et al., 2015), information-processing ability (Nadkarni et al., 2013), etc., and correlations between GMV reductions and diseases including major depression (Abe et al., 2010), chronic pain in trigeminal neuralgia (Obermann et al., 2013), cognitive deficits in dementia (Sanchez-Castaneda et al., 2009), etc. However, we have to note that there are opposed findings (e.g., Li et al., 2018) that challenge an unambiguous positive relation of GMV and health.

The nGMV was derived as an average of standardized gray matter measure for 116 brain regions based on the automated anatomical labeling (AAL) atlas (Tzourio-Mazoyer et al., 2002). Since this measure reflects GMV, we hypothesized that it could vary based on food intake patterns. Therefore, in this study, we investigated the cross-sectional relationship between the nGMV of healthy participants and the results of a brief self-administered diet history questionnaire (BDHQ), which was developed for assessing Japanese consumption frequency of selected foods to estimate the dietary intake of food and beverage items during the preceding month.

## Materials and Methods

### Subjects

One hundred and seventy-one healthy participants (89 females and 82 males), aged 30–69 (mean (M) ± standard deviation (SD): 49.8 ± 8.3 years) were recruited in Kyoto, Japan. This study was approved by the Ethics Committees of Kyoto University (approval number 27-P-13) and performed in accordance with the guidelines and regulations of the institute, respecting the Declaration of Helsinki. All participants gave written informed consent prior to participation, and participant anonymity was preserved.

### Food-Intake Scales

Dietary habits during the preceding month were assessed using a validated BDHQ (Sasaki et al., 2000), which contained queries about the consumption frequency of 56 foods and beverages and nine dishes commonly consumed by the general Japanese population. These Food and beverage items contained in the BDHQ were selected mainly from a food list used in the National Health and Nutrition Survey of Japan (Ministry of Health, Labour and Welfare of Japan, 2008), while standard portion sizes were derived from several recipe books for Japanese dishes (Sasaki et al., 2000). Dietary intake (g/time) was estimated using an *ad hoc* computer algorithm, based on the Standard Tables of Food Composition in Japan (Department of Social and Preventive Epidemiology, School of Public Health, the University of Tokyo, 2008). For instance, consumption of milk/yogurt for a man who perceives consuming less than ordinary man is estimated to be 155 g/time, which is calculated as: 150 g/time (average woman consumption) times 1.15 (the difference of necessary energy between men and women) times 0.9 (individual differences of portion volume: eating much more = 1.2; eating slightly more = 1.1; eating almost the same amount = 1.0; eating slightly less = 0.9; and eating much less = 0.8, compared with normal portion of foods prepared at restaurants) times 1 (frequency: every day more than two times = 2; every day one time = 1; 4 to 6 times for a week = 5/7; two to three times for a week = 2.5/7; one time for a week = 1/7; less than one time for a week = 2/30; no consumption = 0). Using this questionnaire, previous research indicated that higher alcohol consumption or lower calcium intake increased the risk of brain microbleeds (Hara et al., 2013).

### MRI Data Acquisition

All magnetic resonance imaging (MRI) data were collected using a 3-T Siemens scanner (Verio, Siemens Medical Solutions, Erlangen, Germany or MAGNETOM Prisma, Siemens, Munich, Germany) with a 32-channel head array coil. A high-resolution structural image was acquired using a three-dimensional (3D) T1-weighted magnetization-prepared rapid-acquisition gradient echo (MP-RAGE) pulse sequence. The parameters were as follows: repetition time (TR), 1900 ms; echo time (TE), 2.52 ms; inversion time (TI), 900 ms; flip angle, 9°; matrix size, 256 × 256; field of view (FOV), 256 mm; and slice thickness, 1 mm.

### MRI Data Analysis

We developed another index which is based on fractional anisotropy (FA) value of white matter (WM) assessed by diffusion tensor imaging analysis (ITU-T, 2018). However, in the current research, we were focused on nGMV, which was calculated according to our previous study (Nemoto et al., 2017). In summary, gray matter images were segmented from T1-weighted images using Statistical Parametric Mapping 12 (SPM12; Wellcome Trust Centre for Neuroimaging, London, United Kingdom) running on MATLAB R2015b (Mathworks Inc., Sherborn, MA, United States), followed by spatial normalization using diffeomorphic anatomical registration through an exponentiated lie algebra (DARTEL) algorithm (Ashburner, 2007) and modulation to preserve the GM volume. All normalized, segmented, and modulated images were smoothed with an 8-mm full width at half-maximum (FWHM) Gaussian kernel. Additionally, intracranial volume (ICV) was calculated by summing the GM, white matter, and cerebrospinal fluid images for each subject. Proportional GM images were generated by dividing smoothed GM images by ICV to control for differences in whole-brain volume across participants. Using these proportional GM images, images for the mean and standard deviation (SD) across participants were generated. Then, we calculated the nGMV using the following formula: 100 + 15 × (individual proportional GM – mean)/SD. Regional GM quotients were then extracted using an AAL atlas (Tzourio-Mazoyer et al., 2002) and averaged across regions to produce participant-specific nGMV. For reference, all of the 116 regional GM quotients, included in the Supplementary Table S1, were correlated with nGMV (*p* < 0.001), indicating that nGMV reflects regional GMV comprehensively.

### Statistical Analysis

We derived dietary patterns through a principal component analysis of food intake for the 42 items of foods, beverages, and dishes (excluding four overlapping items), using with 10 overlapping animal foods and nine animal dishes alternatively. We used eigenvalues, the scree test, and the interpretability of the factors to determine the number of factors that could be retained. The factors each had an eigenvalue greater than one. The scree plots dropped substantially between the second and the third factors (from 3.50 to 2.88 for the dietary pattern I using animal foods; from 3.46 to 3.09 for the dietary pattern II using animal dishes) and remained similar thereafter (2.54 for the fourth and 2.29 for the fifth factor for the dietary pattern I; 2.26 for the fourth and 2.10 for the fifth factor for the dietary pattern II); thus, we retained two factors (for the dietary pattern I and the dietary pattern II) as are shown in Table 1 (the figures are factor loadings derived by principal-components analysis without rotation). Hereafter, we will call the first and the second factors “vegetable-animal balanced dietary pattern” and “alcohol and animal foods dietary pattern,” respectively. The factor scores for each dietary pattern were calculated by adding the food item intakes weighted by their factor loadings and used as the independent variables to determine nGMV.

**TABLE 1:** Factor loading matrix for major dietary patterns identified by principal component analysis.

	**Dietary pattern I**		**Dietary pattern II**	
	**Vegetable-animal balanced dietary pattern**	**Alcohol and animal foods dietary pattern**	**Vegetable-animal balanced dietary pattern**	**Alcohol and animal foods dietary pattern**
Reduced fat milk and yogurt	*0.037*	–0.151	*0.048*	–0.101
Milk and yogurt	*0.410*	–0.137	*0.420*	–0.160
Egg	*0.370*	–0.046	*0.379*	0.053
Tofu/atsuage^a^	*0.501*	–0.077	*0.500*	–0.023
Natto^b^	*0.339*	0.229	*0.310*	0.176
Potatoes	*0.516*	–0.196	*0.522*	–0.079
Pickled green leafy vegetables	*0.348*	0.177	*0.327*	0.242
Other pickled vegetables	*0.283*	0.165	*0.260*	0.240
Lettuces/cabbage (raw)	*0.597*	–0.030	*0.607*	0.050
Green leafy vegetables	*0.656*	–0.140	*0.666*	–0.118
Cabbage/Chinese cabbage	*0.643*	–0.037	*0.662*	0.022
Carrots/pumpkin	*0.626*	–0.393	*0.682*	–0.293
Japanese radish/turnip	*0.568*	–0.093	*0.596*	0.032
Other root vegetables	*0.582*	–0.218	*0.611*	–0.126
Tomatoes	*0.479*	–0.143	*0.499*	–0.031
Mushrooms	*0.582*	–0.310	*0.628*	–0.250
Seaweeds	*0.677*	–0.033	*0.686*	0.024
Western-type confectioneries	*0.239*	–0.276	*0.245*	–0.105
Japanese-type confectioneries	*0.356*	0.035	*0.348*	0.196
Rice crackers/rice cake/	*0.140*	–0.240	*0.146*	0.029
okonomiyaki^c^				
Ice cream	*0.128*	–0.057	*0.110*	0.072
Citrus fruit	*0.420*	–0.189	*0.435*	–0.113
Persimmons/strawberries/kiwifruit	*0.385*	–0.356	*0.417*	–0.245
Other fruit	*0.406*	–0.215	*0.413*	–0.117
Mayonnaise	*0.353*	–0.081	*0.364*	0.050
Bread	*–0.044*	–0.268	*–0.042*	–0.080
Buckwheat noodles	*0.008*	0.341	*–0.028*	0.409
Japanese wheat noodles	*–0.035*	0.134	*–0.051*	0.257
Chinese noodles	*–0.143*	0.447	*–0.187*	0.528
Pasta	*0.067*	0.194	*0.036*	0.388
Green tea	*0.182*	–0.152	*0.194*	–0.146
Black tea/oolong tea	*–0.035*	–0.078	*–0.037*	–0.075
Coffee	*0.109*	0.113	*0.106*	0.253
Cola drink/soft drink	*–0.176*	0.181	*–0.202*	0.342
100% fruit and vegetable juice	*0.024*	0.103	*0.025*	0.134
Rice	*0.008*	0.227	*0.009*	0.317
Miso soup	0.252	0.218	0.256	0.267
Sake^d^	*0.120*	0.417	*0.097*	0.343
Beer	*0.180*	0.517	*0.135*	0.487
Shochu	*0.178*	0.383	*0.144*	0.334
Whisky	*–0.009*	0.292	*–0.024*	0.373
Wine	*0.289*	0.404	*0.256*	0.309
Chicken	*0.294*	0.340		
Pork/beef	*0.384*	0.343		
Ham/sausage/bacon	*0.185*	0.307		
Liver	*0.075*	0.299		
Squid/octopus/shrimp/shellfish	*0.362*	0.154		
Small fish with bones	*0.328*	*0.450*		
Canned tuna	*0.109*	*0.363*		
Dried fish/salted fish	*0.447*	*0.265*		
Oily fish	*0.420*	*0.330*		
Lean fish	*0.429*	*0.420*		
Sashimi/sushi			*0.255*	*0.332*
Grilled fish			*0.314*	*0.194*
Boiled fish/fish served in pot/fish in soup			*0.347*	*0.148*
Tempura fish			*0.206*	*0.612*
Grilled meat/steak			*0.185*	*0.437*
Hamburg steak/curry/meat sauce			*0.032*	*0.301*
Deep fried meat			*–0.032*	*0.555*
Tempura meat			*0.127*	*0.161*
Boiled meat/meat served in			*0.417*	*–0.122*
pot or bowl/meat in soup				

*^*a*^Deep-fried tofu. ^*b*^Fermented soybeans. ^*c*^Meat/fish and vegetables pancake. ^*d*^Japanese traditional alcoholic beverages. Italicized numbers indicate scores higher than 0.25. I includes 10 animal foods but does not include 9 animal dishes. II includes 9 animal dishes but does not include 10 animal foods.*

To investigate the correlation between the nGMV and various variables, we employed hierarchical regression analysis. We entered the control variables of age, sex, and BMI in Step 1 and the main effects of food and food-pattern variables individually in Step 2 and Step3, respectively. We added these respective variables to the models based on the hypothesis that food and food-pattern variables are closely related to nGMV after adjusting for age, sex, and BMI. Significance level was set at *p* < 0.05. All statistical analyses were performed using IBM SPSS Statistics Version 20 (IBM Corp., Armonk, NY, United States).

## Results

Descriptive statistics of subjects and correlation coefficients between variables are shown in Table 2. nGMV correlated with age (*r* = −0.534, *p* < 0.001) and sex (*r* = 0.388, *p* < 0.001). Moreover, nGMV negatively correlated with 19 of 61 food variables and all the four dietary patterns, whereas none of food items correlated positively with nGMV, indicating high-volume intake may lead to lower nGMV irrespective of the type of foods. Table 3 shows the results of regression analyses. In Step 1, both sex (*R* = 0.656, *b* = 0.393, *p* < 0.001) and age (*R* = 0.656, *b* = −0.531, *p* < 0.001) were significant, indicating that nGMV scores tended to be lower in the male and elderly than the female and younger participants. In addition, BMI (*R* = 0.696, *b* = −0.237, *p* < 0.001) was independently significant after controlling for sex and age, indicating that nGMV scores tend to be negatively associated with obesity.

**TABLE 2:** Descriptive statistics and correlations.

		**Mean**	**SD**	***r*^e^**
1	nGMV	98.006	6.775	
2	Age [years]	49.784	8.296	–0.534^∗∗∗^
3	Sex (male = 1, female = 2)	1.520	0.501	0.388^∗∗∗^
4	BMI	22.997	3.594	–0.396^∗∗∗^
5	Reduced fat milk and yogurt	31.122	60.112	0.034
6	Milk and yogurt	73.058	75.323	0.085
7	Egg	46.310	26.407	−0.157^∗^
8	Tofu/atsuage^a^	50.404	38.780	–0.134
9	Natto^b^	14.012	18.082	–0.048
10	Potatoes	41.396	32.427	0.022
11	Pickled green leafy vegetables	7.322	10.086	–0.224^∗∗^
12	Other pickled vegetables	5.378	8.380	–0.226^∗∗^
13	Lettuces/cabbage (raw)	33.292	22.007	–0.219^∗∗^
14	Green leafy vegetables	34.717	33.450	–0.066
15	Cabbage/Chinese cabbage	36.164	29.011	–0.285^∗∗∗^
16	Carrots/pumpkin	17.022	14.961	–0.027
17	Japanese radish/turnip	15.268	15.129	–0.133
18	Other root vegetables	32.449	23.456	0.020
19	Tomatoes	30.149	28.748	–0.130
20	Mushrooms	10.454	8.833	–0.016
21	Seaweeds	9.465	8.644	–0.123
22	Western-type confectioneries	27.613	24.431	0.059
23	Japanese-type confectioneries	9.029	9.553	–0.092
24	Rice crackers/rice cake/okonomiyaki^c^	15.950	15.530	0.062
25	Ice cream	26.451	30.310	–0.016
26	Citrus fruit	14.196	21.625	–0.019
27	Persimmons/strawberries/kiwifruit	11.850	21.920	0.087
28	Other fruit	31.151	31.909	–0.022
29	Mayonnaise	7.350	4.406	–0.137
30	Bread	43.787	27.012	0.101
31	Buckwheat noodles	15.877	20.405	–0.117
32	Japanese wheat noodles	23.650	23.254	–0.109
33	Chinese noodles	18.231	19.086	–0.065
34	PASTA	17.582	18.424	–0.009
35	Green tea	241.795	225.260	–0.114
36	Black tea/oolong tea	93.024	146.443	–0.014
37	Coffee	241.389	187.690	−0.211^∗^
38	Cola drink/soft drink	61.291	99.383	–0.020
39	100% fruit and vegetable juice	48.827	81.987	–0.134
40	Rice	295.733	186.373	–0.303^∗∗∗^
41	Miso soup	132.370	106.425	–0.263^∗∗∗^
42	Sake^d^	9.638	33.611	−0.172^∗^
43	Beer	89.138	163.968	–0.223^∗∗^
44	Shochu	12.803	30.015	−0.184^∗^
45	Whisky	2.583	12.393	0.023
46	Wine	16.515	53.421	–0.245^∗∗∗^
47	Chicken	33.666	26.643	–0.064
48	Pork/beef	38.990	23.252	–0.138
49	Ham/sausage/bacon	10.241	9.171	–0.066
50	Liver	1.682	7.888	–0.076
51	Squid/octopus/shrimp/shellfish	15.014	12.888	–0.024
52	Small fish with bones	7.877	10.508	−0.197^∗^
53	Canned tuna	3.622	9.481	–0.064
54	Dried fish/salted fish	16.509	14.936	–0.150
55	Oily fish	16.986	14.604	–0.090
56	Lean fish	14.361	13.160	–0.245
57	Sashimi/sushi	27.600	27.107	−0.181^∗^
58	Grilled fish	29.853	22.673	−0.178^∗^
59	Boiled fish/fish served in pot/fish in soup	49.912	45.299	−0.162^∗^
60	Tempura fish	17.534	14.721	−0.194^∗^
61	Grilled meat/steak	20.760	21.371	–0.226^∗∗^
62	Hamburg steak/curry/meat sauce	43.204	29.144	0.069
63	Deep fried meat	30.186	22.601	–0.030
64	Tempura meat	67.549	38.038	–0.032
65	Boiled meat/meat served in pot or bowl/meat	93.535	61.353	0.048
66	Vegetable-animal balanced dietary pattern I	0.000	1.000	–0.228^∗∗^
67	Alcohol and animal foods dietary pattern I	0.000	1.000	–0.307^∗∗∗^
68	Vegetable-animal balanced dietary pattern II	0.000	1.000	–0.207^∗∗^
69	Alcohol and animal foods dietary pattern II	0.000	1.000	–0.341^∗∗∗^

**n* = 171, ^∗^*p* < 0.05, ^∗∗^*p* < 0.01, ^∗∗∗^*p* < 0.001. ^*a*^Deep-fried tofu. ^*b*^Fermented soybeans. ^*c*^Meat/fish and vegetables pancake. ^*d*^Japanese traditional alcohol beverages. ^*e*^Correlation coefficients with nGMV, The units for the values following row 5 are food-intake volumes [g/day].*

**TABLE 3:** Multiple regression analysis of food- or nutrition-intake factors on nGMV.

	**nGMV**															
	**β^a,b^**	***p*-Value**	**β^a,b^**	***p*-Value**	**β^a,b^**	***p*-Value**	**β^a, b^**	***p*-Value**	**β^a, b^**	***p*-Value**	**β^a, b^**	***p*-Value**	**β^a, b^**	***p*-Value**	**β^a, b^**	***p*-Value**
**Step 1**																
**Control variables**																
Age	–0.531	<0.001^***^	–0.520	<0.001^***^												
Sex (male = 1, female = 2)	0.393	<0.001^***^	0.292	<0.001^***^												
BMI			–0.237	<0.001^***^												
R	0.656	<0.001^***^	0.696	<0.001^***^												
R^2^	0.430		0.485													
**Step 2**																
**Control variables**																
Age	–0.567	<0.001^***^	–0.549	<0.001^***^	–0.521	<0.001^***^	–0.505	<0.001^***^								
Sex (male = 1, female = 2)	0.371	<0.001^***^	0.272	<0.001^***^	0.352	<0.001^***^	0.253	<0.001^***^								
BMI			–0.234	<0.001^***^			–0.235	<0.001^***^								
**Main variables**																
Milk and yogurt	0.145	0.015^∗^	0.141	0.014^∗^												
Rice					–0.133	0.030^∗^	–0.129	0.028^∗^								
R	0.677	<0.001^***^	0.710	<0.001^***^	0.781	<0.001^***^	0.707	<0.001^***^								
R^2^	0.459		0.504		0.610		0.500									
**Step 2 (continued)**																
**Control variables**																
Age	–0.542	<0.001^***^	–0.525	<0.001^***^	–0.523	<0.001^***^	–0.509	<0.001^***^	–0.529	<0.001^***^	–0.514	<0.001^***^				
Sex (male = 1, female = 2)	0.372	<0.001^***^	0.281	<0.001^***^	0.372	<0.001^***^	0.279	<0.001^***^	0.373	<0.001^***^	0.280	<0.001^***^				
BMI			–0.223	<0.001^***^			–0.226	<0.001^***^			–0.225	<0.001^***^				
**Main variables**																
Sake	–0.133	0.023^∗^	–0.108	0.057												
Wine					–0.123	0.038^∗^	–0.103	0.072								
Grilled meat/steak									–0.142	0.015^∗^	–0.123	0.029^∗^				
R	0.676	<0.001^***^	0.704	<0.001^***^	0.674	<0.001^***^	0.704	<0.001^***^	0.678	<0.001^***^	0.707	<0.001^***^				
R^2^	0.456		0.496		0.454		0.495		0.459		0.500					
**Step 3**																
**Control variables**																
Age	–0.513	<0.001^***^	–0.503	<0.001^***^	–0.529	<0.001^***^	–0.513	<0.001^***^	–0.516	<0.001^***^	–0.506	<0.001^***^	–0.538	<0.001^***^	–0.521	<0.001^***^
Sex (male = 1, female = 2)	0.393	<0.001^***^	0.285	<0.001^***^	0.319	<0.001^***^	0.226	<0.001^***^	0.395	<0.001^***^	0.285	<0.001^***^	0.268	<0.001^***^	0.189	<0.001^***^
BMI			–0.240	<0.001^***^			–0.231	<0.001^***^			–0.241	<0.001^***^			–0.221	<0.001^***^
**Main variables**																
Vegetable-animal balanced dietary pattern I	–0.052	0.415	–0.031	0.618												
Alcohol and animal foods dietary pattern I					–0.149	0.038^∗^	–0.125	0.072								
Vegetable-animal balanced dietary pattern II									–0.040	0.527	–0.022	0.721				
Alcohol and animal foods dietary pattern II													–0.212	0.003^∗∗^	–0.178	0.011^∗^
R	0.658	<0.001^***^	0.691	<0.001^***^	0.669	<0.001^***^	0.699	<0.001^***^	0.657	<0.001^***^	0.691	<0.001^***^	0.678	<0.001^***^	0.705	<0.001^***^
R^2^	0.433		0.478		0.447		0.489		0.432		0.477		0.460		0.497	

**n* = 171, ^∗^*p* <0.05, ^∗∗^*p* <0.01, ^∗∗∗^*p* <0.001. ^*a*^Standardized regression coefficient. ^*b*^Independent variables were selected using the forced entry method. I includes 10 animal foods but does not include 9 animal dishes. II includes 9 animal dishes but does not include 10 animal foods.*

In Step 2, with age and sex as control variables, milk, and yogurt (*R* = 0.677, *b* = 0.145, *p* = 0.015), rice (*R* = 0.781, *b* = −0.133, *p* = 0.030), sake (*R* = 0.676, *b* = −0.133, *p* = 0.023), wine (*R* = 0.674, *b* = −0.123, *p* = 0.038), and grilled meat/steak (*R* = 0.678, *b* = −0.142, *p* = 0.015) were significantly associated with nGMV, but other food variables were not. However, after including BMI as an additional control variable, only milk and yogurt (*R* = 0.710, *b* = 0.141, *p* = 0.014), rice (*R* = 0.707, *b* = −0.129, *p* = 0.028), and grilled meat/steak (*R* = 0.707, *b* = −0.123, *p* = 0.029) were significantly associated with nGMV, but sake (*R* = 0.704, *b* = −0.108, *p* = 0.057) and wine (*R* = 0.704, *b* = −0.103, *p* = 0.072) were not.

In several cases SD is significantly larger than mean, indicating that some foods were consumed in considerable amounts by some participants, while others did not consume these foods at all (see Supplementary Table S1). In these cases it must be discussed, whether there is an actual linear correlation of the amount of intake with GMV, or whether there is a non-linear function between no consummation and any consummation. Therefore, we alternatively employed dichotomous values (consumption = 1; no consumption = 0) for milk and yogurt, sake, wine, and grilled meat/steak as the independent variables (omitted in the tables but available upon request). However, none of them was significantly associated with nGMV, indicating that small amount of consumption does not influence nGMV.

In step 3, using age and sex as control variables, alcohol and animal foods dietary pattern (I) (*R* = 0.669, *b* = −0.149. *p* = 0.028) and alcohol and animal foods dietary pattern (II) (*R* = 0.678, *b* = −0.212. *p* = 0.003) were significantly associated with a lower nGMV but vegetable-animal balanced dietary pattern (I) (*R* = 0.658, *b* = −0.052. *p* = 0.415) and vegetable-animal balanced dietary pattern (II) (*R* = 0.657, *b* = −0.040. *p* = 0.527) were not. Additionally, using BMI as a control variable, only alcohol and animal foods dietary pattern (II) (*R* = 0.705, *b* = −0.178. *p* = 0.011) was significantly associated with a lower nGMV.

## Discussion

Diet and nutrition play a key role in the promotion and maintenance of good health, as eating healthy can not only lower the risk of developing health problems, but also help manage health issues such as heart disease and diabetes (Gadiraju et al., 2015). Optimal food intake and optimal physical activity has been shown to influence good brain health. However, determination of these “optimal” levels is extremely difficult, made harder due to a lack of objective methods to measure food intake (Dauncey, 2012). In this research, we used the MRI-based nGMV as an objective scale to measure the association between food intake and the whole brain (please note that this study is cross-sectional and the findings in a strict sense report only correlations of nutritional styles with GMV, not cause and effect chains). We analyzed the relationship between the nGMV of healthy participants and the data obtained from a BDHQ. Interestingly, our results indicate higher nGMV in participants partaking a diet rich in milk and yogurt, and lower nGMV in participants partaking “alcohol and animal foods dietary pattern” (high intake of alcohol and animal foods) and a diet rich in rice, sake, wine, grilled meat/steak, alcohol, and animal foods. Thus, consuming more milk and yogurt, and less rice, sake, wine, grilled meat/steak, or alcohol could increase the nGMV, and help stave off brain atrophy. Surprisingly, the other foods and food pattern “vegetable-animal balanced dietary pattern” had no impact on the nGMV, indicating that although balanced vegetables and animal foods may not lead to brain atrophy, they do not help against lower nGMV either.

The result of the positive correlation between milk/yogurt and nGMV reiterates that dairy foods provide several important nutrients beneficial for bone, cardiovascular, and overall health (Rice et al., 2013). Moreover, bioactive components that alter serum lipid levels may be present in milk fat (US Department of Health and Human Services et al., 2010; Rice et al., 2013). Therefore, whereas some studies point to detrimental effects of dairy (Bolland et al., 2011; Goldbohm et al., 2011), majority of research indicates that dairy foods may have beneficial effects on the risks of coronary heart diseases (Rice et al., 2013), cardiovascular diseases (Soedamah-Muthu et al., 2010), elevated blood pressure (Ralston et al., 2012), obesity (Kratz et al., 2013), and type 2 diabetes (Malik et al., 2011; Tong et al., 2011). A study by Miyake et al. (2016) used similar parameters as in the current research, and showed a significant inverse relationship between milk intake during pregnancy and the risk of postpartum depression symptoms (Miyake et al., 2016), which has the potential to lead to a reduced GMV in the anterior cingulate cortex (Grieve et al., 2013), the bilateral caudate nucleus, and the thalamus (Kim et al., 2008). This is because milk is a source of many biologically active substances such as proteins, lipids, carbohydrates, lactose, vitamins, minerals, enzymes, hormones, immunoglobulins, and growth factors, that may confer many health benefits (Hsieh et al., 2015).

Previous research indicates that high intake of processed meat and fried food is positively associated with depression symptoms (Akbaraly et al., 2009), type 2 diabetes, heart failure, obesity, and hypertension (Gadiraju et al., 2015). Reduced intake of meat products is associated with increased total GM volume (Titova et al., 2013b). A meta-analysis of nine studies consisting of 296 alcohol-dependent patients and 359 healthy controls found regional GM atrophy in alcohol-dependent patients in the prefrontal cortex (including the anterior cingulate cortex), the dorsal striatum/insula, and the posterior cingulate cortex consistently across studies (Xiao et al., 2015). Therefore, our current results, implicating high intake of alcoholic beverages and grilled meat/steak or tendency toward alcohol and animal foods dietary pattern in reduction of nGMV are in line with these studies.

Higher consumption of white rice was associated with a significantly increased risk of type 2 diabetes, especially in Asian (Chinese and Japanese) population (Hu et al., 2012). In another study, global loss, and regional alterations in GMV occurred in obese male subjects, suggesting that male subjects with a high BMI are at a greater risk for future decline in cognition or other brain functions (Taki et al., 2008). Similarly, people who were obese had smaller total GMV (Gunstad et al., 2008) or smaller GMV in the bilateral supplementary motor area, bilateral dorsolateral prefrontal cortex, left inferior frontal gyrus and left postcentral gyrus (Brooks et al., 2013) than that in the normal weight or overweight individuals. Therefore, our findings are also in line with these studies, and at the same time present a new observation that white rice is negatively associated with nGMV after controlling for gender, age, and BMI, indicating that high intake of white rice may lead to brain atrophy independent of obesity. A possible reason for this is that high consumption of white rice replaces other foods and thus leads to insufficient intake of essential nutrients for the brain (e.g., thiamine, niacin) (Harper, 2006; Walters et al., 2017).

Mediterranean-type diet (Medi) has gained immense popularity of late. Previous research showed that adherence to Medi was associated with less brain atrophy among older adults and further indicated that higher fish and lower meat intake were the two key elements that contributed to the benefits of Medi on different brain regions, including the GMV (Gu et al., 2015). Our current research replicates the results of this study by showing that lower consumption of meat is associated with greater GMV. However, the association between increased fish consumption and larger GMV was not replicated in our study. Differences in dietary habits or the type of fish consumed between the United States and Japanese populations might be a factor for the discrepancy in the results. Moreover, all fish and shellfish are not necessarily healthy since dried or salted fish are high in salt. Also, the health effects of seafood other than fish are unclear (Nanri et al., 2010).

To summarize, the results of our research indicating that a high intake of milk/yogurt, and a low intake of rice, alcoholic beverages, and grilled meat/steak is associated with higher nGMV, are in line with the published data. Additionally, nGMV, as an objective measure of the association between food intake and the brain, might provide useful information for considering “optimal” food intake for brain health. However, some limitations of the current study should be acknowledged. First, the BDHQ relies on an individual’s capacity to recall their dietary behavior over the past month. Recall bias in dietary behavior could be a systematic bias. Second, this is a cross-sectional study, hampering the possibility of inferring causality between determinant and outcome. Given that dietary habits seem to be relatively invariable throughout life (Bjelland et al., 2013), we might be better able to utilize the associations found in our study which refers to a long-term cumulative effect on brain structure rather than an acute effect from recent consumption. Third, BDHQ is developed for and validated in the Japanese population, which might restrict its generalizability to other countries and their population. It is interesting whether some results – e.g., higher milk intake correlates with higher GMV – can be transferred to a European population with a usually high consummation of milk and milk products. Indeed, according to the data in the Supplementary Table S1, a number of the current research participants did not take any milk at all, which would be less common in Europe. Forth, although we found the dietary pattern which is associated with lower nGMV, we could not find the dietary pattern which is associated with higher nGMV. We only found that a single food, milk/yogurt, is associated with higher nGMV. However, given that foods are consumed in combination and their complex effects are likely to be interactive or synergistic, future research may contribute to the literature by inventing food pattern which is positively associated with nGMV. Fifth, we implicitly related healthiness with the nGMV, which gives a measure of deviation of GMV from an average over a population. However, given the multitude of healthy lifestyles, it must be critically discussed whether it is wise to align healthy lifestyle with a specific brain measure like GMV. Indeed, the interpretation that more GMV is always healthier is an unjustified generalization – there are examples where locally increased gray matter may indicate e.g., presence of psychiatric disease. For instance, decreased GMV was observed in bilateral dorsolateral prefrontal and middle cingulate cortices, while increased GMV was observed in bilateral hippocampus of primary insomnia patients compared with healthy control (Li et al., 2018). In addition, there are other possible definitions of health, considering cardiovascular aspects, absence of low level inflammation indicators, and function of immune response, all of them possibly depending on lifestyle. Therefore, future research is recommended to analyze more comprehensively using these variables in a single model to validate the relation observed in this study.

## Data Availability Statement

All datasets generated for this study are included in the article/Supplementary Material.

## Ethics Statement

The studies involving human participants were reviewed and approved by the Ethics Committees of Kyoto University (approval number 27-P-13). The patients/participants provided their written informed consent to participate in this study.

## Author Contributions

KK wrote the main manuscript text and prepared the tables. YY was responsible for the conceptualization, data curation, funding acquisition, and project administration. Both authors reviewed and edited the manuscript.

## Conflict of Interest

YY was employed by NTT Data Institute of Management Consulting, Inc. The remaining author declares that the research was conducted in the absence of any commercial or financial relationships that could be construed as a potential conflict of interest.

## Supplementary Material

TABLE S1 Data used for regression analyses.

## References

[B1] Abe O. Yamasue H. Kasai K. Yamada H. Aoki S. Inoue H. (2010). Voxel-based analyses of gray/white matter volume and diffusion tensor data in major depression. *Psychiatry Res. Neuroimaging* 181 64–70. 10.1016/j.pscychresns.2009.07.007 19959342

[B2] Akbaraly T. N. Brunner E. J. Ferrie J. E. Marmot M. G. Kivimaki M. Singh-Manoux A. (2009). Dietary pattern and depressive symptoms in middle age. *Br. J. Psychiatry* 195 408–413. 10.1192/bjp.bp.108.058925 19880930 PMC2801825

[B3] Ashburner J. (2007). A fast diffeomorphic image registration algorithm. *Neuroimage* 38 95–113. 10.1016/j.neuroimage.2007.07.007 17761438

[B4] Bjelland M. Brantsæter A. L. Haugen M. Meltzer H. M. Nystad W. Andersen L. F. (2013). Changes and tracking of fruit, vegetables and sugar-sweetened beverages intake from 18 months to 7 years in the Norwegian mother and child cohort study. *BMC Public Health* 13:793. 10.1186/1471-2458-13-793 24103398 PMC3765981

[B5] Bolland M. J. Grey A. Avenell A. Gamble G. D. Reid I. R. (2011). Calcium supplements with or without vitamin D and risk of cardiovascular events: reanalysis of the Women’s Health Initiative limited access dataset and meta-analysis. *BMJ* 342:d2040. 10.1136/bmj.d2040 21505219 PMC3079822

[B6] Brooks S. J. Benedict C. Burgos J. Kempton M. J. Kullberg J. Nordenskjöld R. (2013). Late-life obesity is associated with smaller global and regional gray matter volumes: a voxel-based morphometric study. *Int. J. Obes.* 37 230–236. 10.1038/ijo.2012.13 22290540 PMC3572402

[B7] Croll P. H. Voortman T. Ikram M. A. Franco O. H. Schoufour J. D. Bos D. (2018). Better diet quality relates to larger brain tissue volumes: the rotterdam study. *Neurology* 90 e2166–e2173. 10.1212/WNL.0000000000005691 29769374

[B8] Dauncey M. J. (2012). Recent advances in nutrition, genes and brain health. *Proc. Nutr. Soc.* 71 581–591. 10.1017/S0029665112000237 22716958

[B9] Department of Social and Preventive Epidemiology, School of Public Health, the University of Tokyo (2008). *BDHQ Utilization Manual.* Available at: http://www.nutrepi.m.u-tokyo.ac.jp/dhq/manual/manual.html (accessed July 01, 2019).

[B10] Eres R. Decety J. Louis W. R. Molenberghs P. (2015). Individual differences in local gray matter density are associated with differences in affective and cognitive empathy. *NeuroImage* 117 305–310. 10.1016/j.neuroimage.2015.05.038 26008886

[B11] Espeland M. A. Erickson K. Neiberg R. H. Jakicic J. M. Wadden T. A. Wing R. R. (2016). Brain and white matter hyperintensity volumes after 10 years of random assignment to lifestyle intervention. *Diabetes Care* 39 764–771. 10.2337/dc15-2230 27208378 PMC4839171

[B12] Gadiraju T. Patel Y. Gaziano J. Djoussé L. (2015). Fried food consumption and cardiovascular health: a review of current evidence. *Nutrients* 7 8424–8430. 10.3390/nu7105404 26457715 PMC4632424

[B13] Goldbohm R. A. Chorus A. M. Galindo Garre F. Schouten L. J. van den Brandt P. A. (2011). Dairy consumption and 10-y total and cardiovascular mortality: a prospective cohort study in the Netherlands–. *Am. J. Clin. Nutr.* 93 615–627. 10.3945/ajcn.110.000430 21270377

[B14] Grieve S. M. Korgaonkar M. S. Koslow S. H. Gordon E. Williams L. M. (2013). Widespread reductions in gray matter volume in depression. *Neuroimage Clin.* 3 332–339. 10.1016/j.nicl.2013.08.016 24273717 PMC3814952

[B15] Gu Y. Brickman A. M. Stern Y. Habeck C. G. Razlighi Q. R. Luchsinger J. A. (2015). Mediterranean diet and brain structure in a multiethnic elderly cohort. *Neurology* 85 1744–1751. 10.1212/WNL.0000000000002121 26491085 PMC4653103

[B16] Gu Y. Vorburger R. S. Gazes Y. Habeck C. G. Stern Y. Luchsinger J. A. (2016). White matter integrity as a mediator in the relationship between dietary nutrients and cognition in the elderly. *Ann. Neurol.* 79 1014–1025. 10.1002/ana.24674 27129740 PMC4884180

[B17] Gunstad J. Paul R. H. Cohen R. A. Tate D. F. Spitznagel M. B. Grieve S. (2008). Relationship between body mass index and brain volume in healthy adults. *Int. J. Neurosci.* 118 1582–1593. 10.1080/00207450701392282 18853335

[B18] Hara M. Yakushiji Y. Nannri H. Sasaki S. Noguchi T. Nishiyama M. (2013). Joint effect of hypertension and lifestyle-related risk factors on the risk of brain microbleeds in healthy individuals. *Hypertens. Res.* 36 789–794. 10.1038/hr.2013.26 23575379

[B19] Harper C. (2006). Thiamine (vitamin B1) deficiency and associated brain damage is still common throughout the world and prevention is simple and safe! *Eur. J. Neurol.* 13 1078–1082. 10.1111/j.1468-1331.2006.01530.x 16987159

[B20] Hsieh C. C. Hernández-Ledesma B. Fernández-Tomé S. Weinborn V. Barile D. de Moura Bell J. M. L. (2015). Milk proteins, peptides, and oligosaccharides: effects against the 21st century disorders. *BioMed. Res. Int.* 2015:146840. 10.1155/2015/146840 25789308 PMC4350585

[B21] Hu E. A. Pan A. Malik V. Sun Q. (2012). White rice consumption and risk of type 2 diabetes: meta-analysis and systematic review. *BMJ* 344:e1454. 10.1136/bmj.e1454 22422870 PMC3307808

[B22] Hu F. B. (2002). Dietary pattern analysis: a new direction in nutritional epidemiology. *Curr. Opin. Lipidol.* 13 3–9. 10.1097/00041433-200202000-00002 11790957

[B23] ITU-T (2018). *Recommendation ITU-T H.861.1, Requirements on Establishing Brain Healthcare Quotients. Series H: Audiovisual and Multimedia Systems, E-Health Multimedia Services and Applications – Multimedia e-Health Data Exchange Services.* Available at: https://www.itu.int/rec/T-REC-H.861.1 (accessed July 01, 2019).

[B24] Jacka F. N. Cherbuin N. Anstey K. J. Sachdev P. Butterworth P. (2015). Western diet is associated with a smaller hippocampus: a longitudinal investigation. *BMC Med.* 13:215. 10.1186/s12916-015-0461-x 26349802 PMC4563885

[B25] Killgore W. D. Weber M. Schwab Z. J. DelDonno S. R. Kipman M. Weiner M. R. (2012). Gray matter correlates of Trait and Ability models of emotional intelligence. *Neuroreport* 23 551–555. 10.1097/WNR.0b013e32835446f7 22546702

[B26] Kim M. J. Hamilton J. P. Gotlib I. H. (2008). Reduced caudate gray matter volume in women with major depressive disorder. *Psychiatry Res. Neuroimaging* 164 114–122. 10.1016/j.pscychresns.2007.12.020 18930633 PMC2600594

[B27] Kokubun K. Nemoto K. Oka H. Fukuda H. Yamakawa Y. Watanabe Y. (2018). Association of fatigue and stress with gray matter volume. *Front. Behav. Neurosci.* 12:154. 10.3389/fnbeh.2018.00154 30087602 PMC6066525

[B28] Kratz M. Baars T. Guyenet S. (2013). The relationship between high-fat dairy consumption and obesity, cardiovascular, and metabolic disease. *Eur. J. Clin. Nutr.* 52 1–24. 10.1007/s00394-012-0418-1 22810464

[B29] Li M. Yan J. Li S. Wang T. Wen H. Yin Y. (2018). Altered gray matter volume in primary insomnia patients: a DARTEL-VBM study. *Brain Imaging Behav.* 12 1759–1767. 10.1007/s11682-018-9844-x 29411240

[B30] Malik V. S. Sun Q. van Dam R. M. Rimm E. B. Willett W. C. Rosner B. (2011). Adolescent dairy product consumption and risk of type 2 diabetes in middle-aged women. *Am. J. Clin. Nutr.* 94 854–861. 10.3945/ajcn.110.009621 21753066 PMC3155931

[B31] Ministry of Health, Labour and Welfare of Japan, (2008). *The National Health, and Nutrition Survey in Japan, 2005.* Tokyo: Ministry of Health and Welfare.

[B32] Miyake Y. Tanaka K. Okubo H. Sasaki S. Furukawa S. Arakawa M. (2016). Milk intake during pregnancy is inversely associated with the risk of postpartum depressive symptoms in Japan: the Kyushu Okinawa maternal and child health study. *Nutr. Res.* 36 907–913. 10.1016/j.nutres.2016.06.001 27632910

[B33] Nadkarni N. K. Nunley K. A. Aizenstein H. Harris T. B. Yaffe K. Satterfield S. (2013). Association between cerebellar gray matter volumes, gait speed, and information-processing ability in older adults enrolled in the Health ABC study. *J. Gerontol. A Biol. Sci. Med. Sci.* 69 996–1003. 10.1093/gerona/glt151 24170673 PMC4095927

[B34] Nanri A. Kimura Y. Matsushita Y. Ohta M. Sato M. Mishima N. (2010). Dietary patterns and depressive symptoms among Japanese men and women. *Eur. J. Clin. Nutr.* 64 832–839. 10.1038/ejcn.2010.86 20485303

[B35] Nemoto K. Oka H. Fukuda H. Yamakawa Y. (2017). MRI-based Brain Healthcare Quotients: a bridge between neural and behavioral analyses for keeping the brain healthy. *PLoS One* 12:e0187137. 10.1371/journal.pone.0187137 29077756 PMC5659647

[B36] Obermann M. Rodriguez-Raecke R. Naegel S. Holle D. Mueller D. Yoon M. S. (2013). Gray matter volume reduction reflects chronic pain in trigeminal neuralgia. *Neuroimage* 74 352–358. 10.1016/j.neuroimage.2013.02.029 23485849

[B37] Pelletier A. Barul C. Féart C. Helmer C. Bernard C. Periot O. (2015). Mediterranean diet and preserved brain structural connectivity in older subjects. *Alzheimers Dement* 11 1023–1031. 10.1016/j.jalz.2015.06.1888 26190494

[B38] Porter T. M. (2004). *Karl Pearson: The Scientific Life in a Statistical Age.* Princeton, NJ: Princeton University Press.

[B39] Raji C. A. Erickson K. I. Lopez O. L. Kuller L. H. Gach H. M. Thompson P. M. (2014). Regular fish consumption and age-related brain gray matter loss. *Am. J. Prev. Med.* 47 444–451. 10.1016/j.amepre.2014.05.037 25084680 PMC4171345

[B40] Ralston R. A. Lee J. H. Truby H. Palermo C. E. Walker K. Z. (2012). A systematic review and meta-analysis of elevated blood pressure and consumption of dairy foods. *J. Hum. Hypertens.* 26 3–13. 10.1038/jhh.2011.3 21307883

[B41] Rice B. H. Quann E. E. Miller G. D. (2013). Meeting and exceeding dairy recommendations: effects of dairy consumption on nutrient intakes and risk of chronic disease. *Nutr. Rev.* 71 209–223. 10.1111/nure.12007 23550782 PMC3644863

[B42] Sanchez-Castaneda C. Rene R. Ramirez-Ruiz B. Campdelacreu J. Gascon J. Falcon C. (2009). Correlations between gray matter reductions and cognitive deficits in dementia with Lewy Bodies and Parkinson’s disease with dementia. *Mov. Disord.* 24 1740–1746. 10.1002/mds.22488 19569130

[B43] Sasaki S. Ushio F. Amano K. Morihara M. Todoriki T. Uehara Y. (2000). Serum biomarker-based validation of a self-administered diet history questionnaire for Japanese subjects. *J. Nutr. Sci. Vitaminol.* 46 285–296. 10.3177/jnsv.46.285 11227800

[B44] Soedamah-Muthu S. S. Ding E. L. Al-Delaimy W. K. Hu F. B. Engberink M. F. Willett W. C. (2010). Milk and dairy consumption and incidence of cardiovascular diseases and all-cause mortality: dose-response meta-analysis of prospective cohort studies. *Am. J. Clin. Nutr.* 93 158–171. 10.3945/ajcn.2010.29866 21068345 PMC3361013

[B45] Taki Y. Hashizume H. Sassa Y. Takeuchi H. Asano M. Asano K. (2010). Breakfast staple types affect brain gray matter volume and cognitive function in healthy children. *PLoS One* 5:e15213. 10.1371/journal.pone.0015213 21170334 PMC2999543

[B46] Taki Y. Kinomura S. Sato K. Inoue K. Goto R. Okada K. (2008). Relationship between body mass index and gray matter volume in 1,428 healthy individuals. *Obesity* 16 119–124. 10.1038/oby.2007.4 18223623

[B47] Titova O. E. Ax E. Brooks S. J. Sjögren P. Cederholm T. Kilander L. (2013a). Mediterranean diet habits in older individuals: associations with cognitive functioning and brain volumes. *Exp. Gerontol.* 48 1443–1448. 10.1016/j.exger.2013.10.002 24126083

[B48] Titova O. E. Sjögren P. Brooks S. J. Kullberg J. Ax E. Kilander L. (2013b). Dietary intake of eicosapentaenoic and docosahexaenoic acids is linked to gray matter volume and cognitive function in elderly. *Age* 35 1495–1505. 10.1007/s11357-012-9453-3 22791395 PMC3705118

[B49] Tong X. Dong J. Y. Wu Z. W. Li W. Qin L. Q. (2011). Dairy consumption and risk of type 2 diabetes mellitus: a meta-analysis of cohort studies. *Eur. J. Clin. Nutr.* 65 1027–1031. 10.1038/ejcn.2011.62 21559046

[B50] Tzourio-Mazoyer N. Landeau B. Papathanassiou D. Crivello F. Etard O. Delcroix N. (2002). Automated anatomical labeling of activations in SPM using a macroscopic anatomical parcellation of the MNI MRI single-subject brain. *Neuroimage* 15 273–289. 10.1006/nimg.2001.0978 11771995

[B51] US Department of Health and Human Services, US Department of Agriculture, and US Dietary Guidelines Advisory Committee (2010). *Dietary Guidelines for Americans, 2010*, 7th Edn, Washington, DC: Government Printing Office.

[B52] Walters M. Hackett K. Caesar E. Isaacson R. Mosconi L. (2017). Role of nutrition to promote healthy brain aging and reduce risk of Alzheimer’s disease. *Curr. Nutr. Rep.* 6 63–71. 10.1007/s1366

[B53] Witte A. V. Kerti L. Hermannstädter H. M. Fiebach J. B. Schreiber S. J. Schuchardt J. P. (2013). Long-chain omega-3 fatty acids improve brain function and structure in older adults. *Cereb. Cortex* 24 3059–3068. 10.1093/cercor/bht163 23796946

[B54] Xiao P. Dai Z. Zhong J. Zhu Y. Shi H. Pan P. (2015). Regional gray matter deficits in alcohol dependence: a meta-analysis of voxel-based morphometry studies. *Drug Alcohol Depend.* 153 22–28. 10.1016/j.drugalcdep.2015.05.030 26072220

